# Unusual Differential Diagnosis of Upper Abdominal Pain

**DOI:** 10.1155/2009/817052

**Published:** 2009-02-16

**Authors:** Lanthaler Monika, Grissmann Thomas, Schwentner Lukas, Nehoda Hermann

**Affiliations:** ^1^Department of General and Transplant Surgery, Innsbruck Medical University Hospital, Anichstrasse 35, 6020 Innsbruck, Austria; ^2^Department of General Surgery, Town Hospital Kitzbühel, Hornweg 28, 6370 Kitzbühel, Austria; ^3^Department of General Surgery, Hospital of St. Johann, Bahnhofstrasse 14, 6380 St. Johann, Tyrol, Austria

## Abstract

We here present an interesting unusual case of upper abdominal pain. The patient was a 38-year-old man, who was admitted to our hospital complaining of right upper quadrant pain caused by a toothpick that perforated the anterior gastric wall and penetrated segment I of the liver. After endoscopic removal and an initially uneventful course, computed tomography revealed a perigastric abscess that was treated by repeated gastroscopic rinsing via an endoscopically placed catheter. After another three uneventful weeks, a liver abscess with minor tendency to constrict the portal vein was diagnosed, and a segment I liver resection together with abscess drainage was performed. The peculiarity of this case is the rarity of toothpick ingestion and gastric perforation in a young and healthy white Caucasian followed by development of a liver abscess after primary uneventful endoscopic removal. In light of this case, gastric perforation due to ingested foreign bodies such as toothpicks can be considered a rare cause of upper abdominal pain.

## 1. Introduction

Most accidentally ingested foreign bodies pass through the gastrointestinal tract without difficulty. In general, perforation is very rare and occurs in less than 1% of patients [1, 2].

We here present a rare case in which an ingested toothpick perforated the anterior gastric wall and penetrated segment I of the liver, resulting in hepatic abscess formation.

## 2. Case Report

A 38-year-old man was admitted to our hospital with a three-month history of upper abdominal pain. He complained of pain consistent with gastritis, loss of appetite, and minor weight loss, but could not remember a particular situation or time when the pain first started. Gastroscopic findings from a different institution were unremarkable. Abdominal examination revealed right upper quadrant pain with neither guarding nor rebound.

Laboratory examination showed a white cell count of 12 200 g/dL and a C-reactive protein of 9.69 g/dL. Sonography and computed tomography revealed a circular swelling of the anterior gastric wall up to 21 mm in diameter and an inflammatory process reaching into the liver, but did not identify a foreign body.

To exclude the tentative radiologic diagnosis of an MALT lymphoma or a gastric carcinoma, regastroscopy was performed.

**Figure 1:**
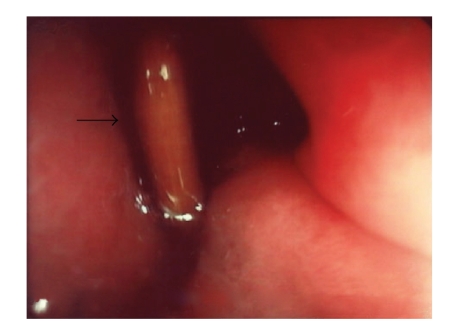
Gastroscopy: toothpick (black arrow) perforating the anterior gastric wall.

**Figure 2:**
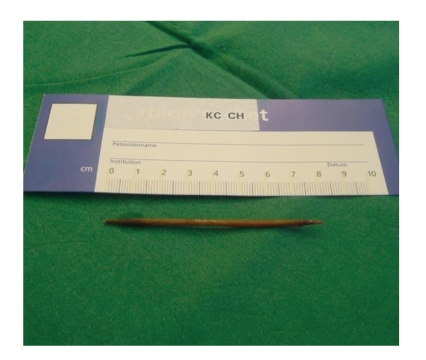
Ingested toothpick.

This time a toothpick perforating the anterior gastric wall was detected that extended half of its length in the direction of the liver (see Figure 1). The toothpick was removed endoscopically using a grasper for extricating foreign bodies (see Figure 2). The esophagus was unremarkable.

After this procedure, the patient experienced immediate pain relief and his course was uneventful thereafter. Control gastroscopy performed 24 hours after removal of the toothpick was normal. Antibiotic therapy with piperacillin/tazobactam and high-dose therapy of pantoprazole were initiated. Abdominal X-ray was unremarkable, and laboratory signs of inflammation decreased. Four days later, the patient was discharged.

**Figure 3:**
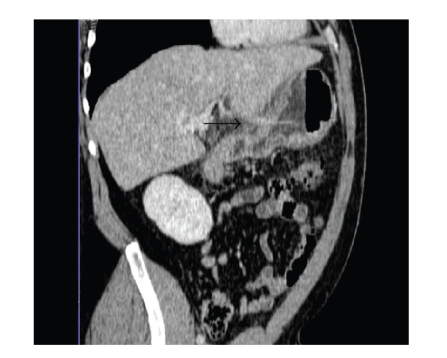
Computed tomography image with a pattern possibly revealing a toothpick (black arrow).

Once the diagnosis was established, a second evaluation of the CT scan revealed a pattern possibly characteristic of a toothpick (see Figure 3).

After discharge, the patient was closely monitored in outpatient check-ups. Antibiotic therapy with cefuroxime was maintained for one more week, and the patient's course was uneventful for two weeks.

Thereafter, the patient again presented with right upper quadrant pain, and laboratory findings indicated severe inflammation. Computed tomography revealed a perigastric abscess that was successfully treated by gastroscopic rinsing. A catheter was endoscopically inserted in the perigastric abscess several times, and through this catheter we rinsed the abscess cavity every time the catheter was introduced. Since this was a unique case, the method was experimental. Treatment of such an abscess using antibiotics alone would have been ineffective. Before finally deciding to operate, we wanted to try gastroscopic rinsing, because it would be much less invasive than an operation and the location of the abscess was optimal for such treatment.

After gastroscopic rinsing, the patient was symptom free and showed no signs of inflammation. Moreover, computed tomography was unremarkable, so that we could reasonably presume at that time that treatment was successful.

After another three uneventful weeks, the patient was admitted again. Once more he complained of right upper quadrant pain, and this time laboratory findings showed leukocytosis to be 18 000 g/dL and C-reactive protein 60 g/dL. Blood cultures remained sterile. Computed tomography revealed a liver abscess mainly in the lobus caudatus with a slight tendency to constrict the portal vein. The abscess was riddled with septa, so that puncture was not possible. Antibiotic therapy with piperacillin/tazobactam was initiated again, and an operative abscess drainage and segment I resection through laparotomy finally performed. The abscess was 6 cm in diameter, and anatomical resection of the entire caudate lobe was necessary. The microbiological findings revealed *Streptococcus milleri* for the abscess material. The intraoperatively placed drain was left in place for five days. The patient's subsequent course was uneventful, and he was discharged symptom free after two more weeks. The patient has remained symptom free until today.

## 3. Discussion

As described in the literature, the majority of foreign bodies transit through the gastrointestinal system without causing complications. The esophagus and the colon are the two main localizations at the highest risk for perforation. The literature contains only few reports of perforation of the gastric wall, usually involving peritonitis that is subsequently treated by laparoscopy or laparotomy [3–5].

From this case, we see that the initially benign course of a gastric perforation due to a toothpick can ultimately become a severely dangerous situation, as when our patient developed a liver abscess. If such an abscess cannot be drained percutaneously because of septa or adverse localization, immediate surgical removal is mandatory to prevent a life-threatening situation like a portal vein thrombosis.
